# Supplementary material for: Phytoplankton community structuring in the absence of resource-based competitive exclusion
Source: PLoS One. 2022 Sep 16;17(9):e0274183. doi: 10.1371/journal.pone.0274183 (PMC9481051; doi:10.1371/journal.pone.0274183)
Supplement: S1 File — (PDF) [file pone.0274183.s001.pdf]

Supporting Information for Behrenfeld et al "Phytoplankton community structuring in the absence of resource-based competitive exclusion"

Note 1: For this size distribution, the differential number concentration, *N(d)* [cells ml^{-3} μm^{-1}] is given by $N(d) = N_0 \left(\frac{d}{d_0}\right)^{-\xi}$, which when integrated between d_{min} and d_{max} gives equation 2.

Note 2: For these modeled populations and assuming *Prochlorococcus* to span a size range of 0.6 to 0.8 μm diameter, the value for N_0 ranges from 2.9×10^{-5} to 1.8×10^{-4} cells ml^{-1} μm^{-1} and can be calculated as: $N_0 = \frac{n\,(1-\xi)}{(0.8^{1-\xi} - 0.6^{1-\xi})}$

Note 3: Note that an I_r term would also need to be added to equation 5 if *P* had been measured as net oxygen production in Fig. 2A, rather than ^{14}C uptake.

Note 4: The I_r term appears in equation 6 because catabolism of carbon products ultimately results in the respiratory production of CO_2, which can isotropically diffuse through the cell and across the outer membrane. In contrast, catabolism of nutrient-containing molecules generally does not proceed to inorganic forms, but rather to intermediate or charged forms that are readily re-assimilated into new products and unlikely to be lost from the cell in significant quantities. For example, nitrogen-containing molecules are degraded to simple amino acids, ammonium, or urea. Accordingly, a term analogous to I_r is not included in equation 8.

Note 5: Note that the '10,000' factors in equations 12a and 12b are for units conversion. The original relationships were developed with diameter in units of cm and velocities in cm s^{-1}. In the current manuscript, diameters have units of μm and velocities have units of μm s^{-1}.

Note 6: Steady-state solutions for the ith phytoplankton and zooplankton concentrations for a given S_∞ can be computed directly (i.e., without running the model over time) for equations 18a and 18b as: $\left(P_i^N, Z_i^N\right) = \left(\frac{g_3}{g_1 g_2 f_i} + \frac{g_4 \mu_i}{g_1^2 g_2 f_i}, \frac{\mu_i}{g_1 f_i}\right)$.

Note 7: The chosen threshold defining whether a modeled phytoplankton species is extant or extinct is intended to be conservative. To put this threshold in perspective, we expect large phytoplankton to be very rare (but not extinct) in oligotrophic waters. If we assume that *Prochlorococcus* has a cell abundance of ~10^5 cell ml^{-1} and that the phytoplankton size distribution has a steep slope of -5.3, then the abundance 135 µm cells (the largest size class represented in our model) would be ~1 cell per 10 m^3 and its contribution to total phytoplankton biomass would be ~0.0008%, which exceeds our 0.0001% threshold defining whether a modeled species is extant or extinct.
